# Collaborative challenges during transition of patients with severe depression from secondary mental health services to primary care and opportunities for improvement - a qualitative study

**DOI:** 10.3389/fpsyt.2025.1553930

**Published:** 2025-10-01

**Authors:** Anne Sofie Aggestrup, Marius Brostrøm Kousgaard, Klaus Martiny, Annette Sofie Davidsen

**Affiliations:** ^1^ Copenhagen Affective Disorder Research Center (CADIC), Psychiatric Center Copenhagen, Copenhagen, Denmark; ^2^ The Research Unit for and Section of General Practice, Department of Public Health, University of Copenhagen, Copenhagen, Denmark; ^3^ Department of Clinical Medicine, University of Copenhagen, Copenhagen, Denmark

**Keywords:** depression, mental health service, general practice, municipality, primary care, secondary care, qualitative methods

## Abstract

**Introduction:**

Patients with severe depression are treated across primary and secondary healthcare and receive employment support from municipal caseworkers (CWs). However, collaboration during the transition from outpatient secondary mental health services to primary care is often insufficient, increasing the risk of relapse. Limited knowledge exists on how health professionals (general practitioners (GPs), mental health professionals (MHPs) and social medicine physicians), CWs, and patients perceive barriers – and, in particular, how collaboration could be improved and relapse prevented. We aimed to explore barriers to cross-sectoral collaboration during the transition of patients with severe depression from outpatient secondary mental health services to primary care, and to generate ideas from health professionals, CWs, and patients to enhance this collaboration and contribute to preventing relapse.

**Materials and methods:**

This qualitative study included fieldwork observations, interviews, focus groups, and workshops with 25 health professionals, seven CWs, and four recently discharged patients. Data was analysed using Interpretative Phenomenological Analysis and descriptive methods.

**Results:**

Four themes were found: Insufficient communication from mental health services caused challenges for GPs in the handover phase; Different goals between professionals resulted in mutual mistrust and blame; Patients felt left on their own after discharge due to a lack of coordination and support services and; Ideas for improving cross-sectoral collaboration. Collaboration across sectors was impeded by low relational coordination, including divergent goals, unclear roles, mutual mistrust and limited communication. An asymmetry in dependence between sectors was evident: GPs and CWs were dependent on information from MHPs during and after outpatient treatment, whereas MHPs were neither aware of this need nor expected ongoing updates. Patients felt vulnerable post-discharge, with little support, and the burden of navigating a complex healthcare system on their own. To improve collaboration, participants suggested online planning meetings, a coordinating liaison or care manager, and a smartphone application for patients to monitor their mental health and signs of relapse. Flexible return to work solutions were also emphasised.

**Discussion:**

Cross-sectoral collaboration in the care of severe depression is challenged by structural and relational barriers, leaving patients vulnerable and at increased risk of relapse during transitions between sectors. Strengthening coordination through shared planning, clearer roles, a coordinating liaison or care manager, digital tools, and flexible return-to-work solutions may ensure continuity and prevent relapse of depression.

## 1 Introduction

Severe depression is a prevalent and recurrent mental health disorder and is ranked among the leading causes of global disability (1). It is considered a chronic condition due to its high relapse rates, with the likelihood of recurrence increasing after each depressive episode (2, 3). The risk of recurrence escalates from 40-60% after one depressive episode to 90% after three depressive episodes (3, 4). The mean time to recurrence following an initial episode is approximately three years, and subsequent episodes often appear within 1 to 1.5 years, particularly during the initial months following recovery (4).

In many countries, up to 90% of patients with depression are managed exclusively by general practitioners (GPs) (5, 6). These cases predominantly involve mild to moderate depressive episodes, which can generally be managed within primary care, whereas specialised services are required for severe cases. Patients with severe depression are therefore often treated within specialised mental health services before transitioning back to general practice. However, this transition between sectors is frequently characterised by insufficient cross-sectoral collaboration (7, 8). In addition, many therapeutic interventions utilised in mental health services fall outside the possibilities in primary care and are therefore not continued in general practice (9, 10). It has been shown that GPs with better access to specialist support demonstrate increased skills, knowledge, and confidence in managing mental disorders (11–13). Nevertheless, GPs report limited access to support from mental health professionals (MHPs) (9, 14–19). Consequently, many patients remain at risk of relapse after discharge from mental health services to general practice because they are still in the recovery process and without optimal support options (20–22).

Insufficient coordination between sectors may result in discontinuity of treatment and support when patients transition from specialised care to primary care. Such treatment gaps are risk factors for relapse, particularly in the vulnerable early recovery phase, where continuity and systematic follow-up are crucial (23, 24). Thus, inadequate cross-sectoral collaboration undermines efforts to prevent relapse. Therefore, continuous treatment and cross-sectoral collaboration are essential for improving recovery and preventing relapse. Enhancing this cross-sectoral collaboration and ensuring continuity of care through organisational initiatives has been a longstanding priority for policymakers (25). Similar issues regarding cross-sectoral collaboration and patient transition have been reported in countries with strong primary care systems, such as the UK, the Netherlands, and other Nordic countries (26–29), highlighting the broader relevance of studying barriers and opportunities for improving collaboration in patients with severe depression.

Several initiatives, such as collaborative care models and primary care support programs, have been introduced in different countries to strengthen the management of depression and facilitate collaboration across sectors (30, 31). These initiatives, initially developed in the United States, are characterised by a multi-professional approach to patient care, a structured management plan, scheduled patient follow-up, and enhanced interprofessional communication (32). Collaborative care models typically involve a GP, a care manager, and a psychiatrist, with the care manager being responsible for delivering care under the supervision of a psychiatrist (33). Systematic reviews have shown that this type of care leads to better outcomes in depression, with benefits lasting up to five years (33, 34). Trials like CADET (35) and COINCIDE (36) in the United Kingdom (UK), and the Collabri Flex trial in Denmark (31), have shown significant positive effects. However, these studies have primarily targeted patients with mild to moderate depression treated in primary care settings (37), although many participants in practice presented with severe depression (31). They have also not included patients who were hospitalised with severe depression, nor involved caseworkers from municipal jobcentres who provide employment support to these patients. It is therefore essential to consider the health and social care system as a whole to promote cross-sector collaboration and to include patients with severe depression in the models, ensuring that they cover the whole spectrum of depression. In addition, not all of these models have been implemented in practice after the project period, as is the case with the Collabri Flex model in Denmark (31).

Therefore, this study aimed to explore barriers to cross-sectoral collaboration during the transition of patients with severe depression from outpatient secondary mental health services to primary care, and to generate ideas from health professionals (GPs, MHPs, and social medicine physicians), CWs, and patients to enhance this collaboration and contribute to preventing relapse.

## 2 Materials and methods

This study represents the initial phase of a larger project aimed at improving recovery in patients treated in secondary mental health services for severe depression who are subsequently transferred to primary care.

We employed a qualitative phenomenological approach, utilising multiple data collection methods, including fieldwork observations, interviews (individual and group), focus groups, and workshops involving MHPs, GPs, CWs, and patients. Data were analysed using Interpretative Phenomenological Analysis (IPA) and a descriptive approach for parts of the data. Data collection and analysis were conducted iteratively, with insights from each stage informing the subsequent data collection.

Within the Danish healthcare system, people with depression are referred to as *‘patients’*, whereas in the social services, they are termed *‘citizens’*. In the results section, we use the participants’ terminology; however, the term *‘patient’* is used consistently throughout the remainder of the article.

### 2.1 Setting

The Danish healthcare system is primarily tax-funded, with approximately 84% of costs covered by taxes. This ensures free access to most healthcare services for all citizens. The system operates across three administrative and political levels: the state, five regions, and 98 municipalities. The regions are responsible for managing hospitals, GPs, and mental health services (38–42). The municipalities are responsible for implementing the labour market policy, which is administered by municipal jobcentres. In this article, municipalities are referred to as *‘social services.’*

The healthcare system is divided into a primary and secondary sector. The primary sector – typically the first point of contact - includes GPs and municipalities. GPs act as gatekeepers and may refer patients to the secondary sector for more specialised treatment such as hospital care or consultations with private practising specialists (38–42). Patients with severe depression are typically on sick leave and receive support from a municipal jobcentre to facilitate a return to work. In some cases, they may be referred to a Social Medicine Department, which provides assessments and advice related to sick leave, flexible employment, and disability pensions.

### 2.2 Participants, eligibility, and recruitment

In total, 25 health professionals (17 females and eight males), seven CWs (six females and one male), and four patients (two females and two males) participated. The ages of the health professionals and CWs ranged from 32 to 79 years. Patients were between 48 and 64 years old (**Table 1**).

**Table 1:** Participant characteristics.

Type of professional, id number	Gender (f/m)	Age (years old)	Duration (rounded minutes)
Individual interviews (conducted between 2022 – 2023)			
GP 1	F	62	50
GP 2	F	53	17
GP 3	F	53	52
Psychiatrist nurse 1	F	53	40
Psychiatrist nurse 2	F	50	37
Psychiatrist nurse 3	F	43	38
Psychiatrist	F	79	50
Group interview (held in 2022)			
Social Medicine Physician 1	F	54	62
Social Medicine Physician 2	F	42	62
Social Medicine Physician 3	F	49	62
Focus Group 1 (held in 2022)			
Caseworker 1	F	47	109
Caseworker 2	F	50	109
Caseworker 3	M	32	109
Focus Group 2 (held in 2022)			
GP 1	F	62	120
GP 2	M	66	120
GP 3	M	62	120
Psychiatrist nurse	F	53	120
Psychiatrist 1	F	71	120
Psychiatrist 2	M	76	120
Practicing Psychiatrist	M	72	120
Social medicine physician	F	61	120
Caseworker 1	F	48	120
Caseworker 2	M	33	120
Workshop 1 (held in 2024)			
GP 1	F	47	240
GP 2	F	64	240
GP 3	F	61	240
GP 4	M	50	240
GP 5	M	70	240
Psychiatrist 1	F	55	240
Psychiatrist 2	F	46	240
Psychiatrist 3	M	63	240
Psychiatrist 4	M	38	240
Caseworker 1	F	35	240
Caseworker 2	F	52	240
Caseworker 3	F	64	240
Caseworker 4	F	33	240
Caseworker 5	F	66	240
Caseworker 6	M	34	240
Workshop 2 (held in 2024)			
Patient 1	F	48	120
Patient 2	F	56	120
Patient 3	M	64	120
Patient 4	M	56	120

Professional participants were recruited by the first author (ASA) and were eligible if employed in the region where the study took place. *Psychiatric nurses* were recruited through convenience sampling, as all three nurses available at the outpatient clinic where patients received treatment were invited to participate. *GPs and psychiatrists* were selected using variation sampling and contacted via email, phone, or direct approaches in their clinics. Recruitment of GPs and psychiatrists was continuous to ensure maximum variation in gender, age, and practice type or workplace. *Social medicine physicians and CWs* were selected using convenience sampling. Their manager initially suggested them and was subsequently contacted by ASA via email.

Eight of the contacted GPs declined to participate, primarily due to a lack of time. A few did not respond to follow-up contact, and one explained her refusal due to plans to sell the practice and retire within the following six months. Only female GPs agreed to participate in the individual interview, whereas both male and female GPs took part in Focus Group 2 and Workshop 1. All the eligible psychiatric nurses were female. A total of eight psychiatrists participated, recruited from various outpatient clinics across the region. Three psychiatrists were unavailable to participate: one could not schedule time for an individual interview, another was unable to attend Workshop 1, and a third, who had initially agreed to participate in Workshop 1, fell ill on the day of the workshop. Among the participating psychiatrists, one was male and two were female; the 79-year-old psychiatrist was still in active clinical practice at the time of the interview.

*Patients were recruited in two stages.* First, the third author (KM) identified eligible patients to ensure variation in age and gender. Additionally, they had to possess the mental capacity to complete a two-hour workshop. Interested patients were then contacted by ASA, who provided detailed oral and written information about the study via phone and email. Eligibility criteria for patients were: age ≥ 18, Danish-speaking, prior inpatient and outpatient treatment for severe depression based on ICD-10 (43), and discharged from intensive outpatient care within the past six months. Exclusion criteria were current psychotic depression, bipolar disorder, or alcohol/substance abuse. Comorbid anxiety was permitted. Five patients agreed to participate in Workshop 2. However, one patient had to withdraw on the day of the workshop, as he felt too vulnerable to participate.

All participants received oral and written information about the study before deciding to participate, and informed consent was obtained from all.

### 2.3 Data collection

**Figure 1:**
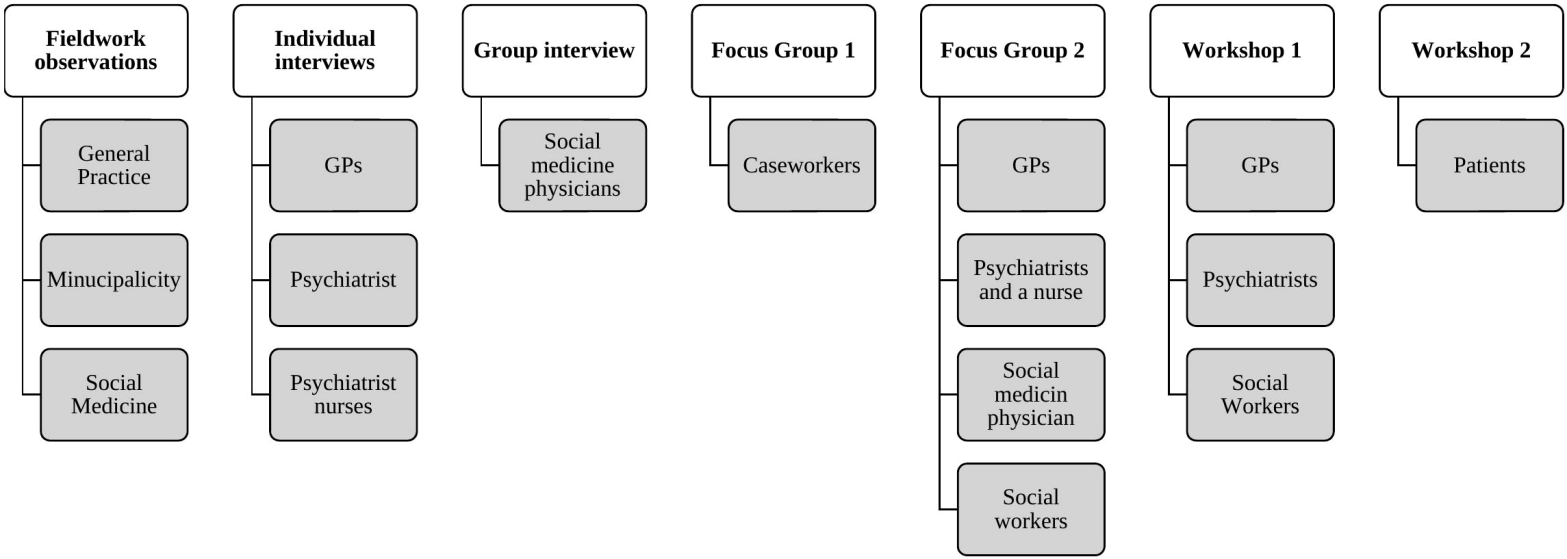
Data collection.

Data were collected from March 2022 to January 2024 in the Capital Region in Denmark and involved fieldwork observations, interviews (individual and group), and workshops (**Figure 1**).

#### 2.3.1 Fieldwork observations

To gain deeper insight into the context of the Danish healthcare system, as well as the daily work of health professionals and CWs, including their interactions with patients and collaboration with colleagues and other professionals across sectors, ASA conducted overt fieldwork observations at:

- Three general practice clinics in Greater Copenhagen
- The Department of Social Medicine, Frederiksberg Hospital
- The largest jobcentre in the Municipality of Copenhagen

The observations were carried out over one day at each site, with field notes taken throughout.

#### 2.3.2 Interviews (individual and group)

Individual interviews and a single group interview were conducted using a semi-structured interview guide to ensure that all relevant topics were covered (44). The guide was developed based on the literature, discussions with the interprofessional project group, and findings from the study of patients’ perspectives (45). It was adapted to the different professional groups to reflect their daily practice, while covering the same overarching topics, including experiences of 1) day-to-day practice and encounters with patients with severe depression, 2) collaboration with other professionals across sectors, and 3) proposals for a new intervention to improve cross-sector collaboration (the interview guide for GPs has been included as a **Supplementary File**).

*Individual interviews* were conducted with one psychiatrist, three psychiatric nurses, and three GPs, all of whom had direct treatment responsibility for the patient group. MHPs manage patients during outpatient treatment, while GPs take over responsibility shortly after patients are discharged from outpatient mental health services. The individual interviews were held at an intensive outpatient mental health service or general practice clinics. The individual interviews ranged from 17 to 51 minutes, with an average duration of 40 minutes (**Table 1**).

*An online group interview* was conducted with social medicine physicians. This interview was added during the project based on a recommendation from the steering group to ensure their perspective was included. Since social medicine physicians are involved in only some instances and do not have regular daily contact with the patient group, they were interviewed as a single group. The group interview lasted one hour.

The individual interviews were audio-recorded, and the group interview was video-recorded. Notes were taken during the interviews. All interviews were conducted and transcribed verbatim by ASA.

#### 2.3.3 Focus groups

Two focus groups were conducted to facilitate dynamic interaction among participants (44). Focus Group 1 was mono-professional, involving CWs, and was held at an outpatient mental health service. Focus Group 2 was cross-sectoral, including 10 health professionals and CWs, and took place in an undisturbed room at the University of Copenhagen. ASA facilitated both groups, with the last author (ASD) assisting in Focus Group 2. Both groups lasted two hours, were video recorded and transcribed by ASA.

#### 2.3.4 Workshops

Two workshops were held to generate ideas about the content, format, and delivery of efforts aimed at improving collaboration. Workshop 1 was cross-sectoral with 16 health professionals and CWs, and Workshop 2 involved four patients. Participants in both workshops worked with sticky notes, notes on regular paper, and notes or drawings on whiteboards.

In Workshop 1, held at the municipal jobcentre, participants worked on generating ideas for a collaborative intervention. ASD led the workshop, which lasted four and a half hours. ASA took detailed notes, and participants’ writings and drawings were preserved.

In Workshop 2, which was held in a neutral group room in the research unit, where patients had previously received group therapy as part of their outpatient mental health treatment, participants were asked to share their reflections on the model proposed by the health professionals and CWs and were encouraged to suggest additional recommendations. Patients who participated in the workshop had been discharged from the outpatient mental health clinic a few months before their participation (between 1 and 6 months prior). ASA led the workshop, which lasted two hours, with KM taking detailed notes.

### 2.4 Researchers roles

The authors represented a diverse range of educational backgrounds and research approaches. ASA is a registered nurse and health sciences graduate and is currently a PhD student. She has received training in qualitative interview and analysis methods. Supported by ASD, she conducted the interviews and did the initial data analysis, which was discussed throughout the process with ASD. ASD is a GP with expertise in psychiatry and psychotherapy, and many years of experience in qualitative research and phenomenology. MBK, a political scientist and associate professor with expertise in implementation science and qualitative research, actively contributed to the analysis, particularly in the discussion and interpretation of themes. KM, professor of clinical psychiatry, has no previous expertise in qualitative research but contributed actively to the reflection on and interpretation of the results.

### 2.5 Data analysis

IPA was applied to the analysis of the interviews and focus groups, allowing for a detailed examination of participants’ lived experiences (46). To facilitate systematic coding and organisation of these data, the qualitative data analysis software NVivo 14 was used. Data from workshops were analysed using a descriptive analysis approach. ASA had the primary responsibility for conducting the analysis, while ASD guided the process and contributed through close collaboration during the entire analysis and subsequent refinement.

The IPA analysis began by listening to the recordings and *reading* the transcripts multiple times to get an overall impression. Next, we identified how participants understood the phenomenon of ‘cross-sector collaboration’ and created *exploratory notes* and comments on each transcript. In the third step, *constructing experiential statements*, we summarised points from the notes and comments, reflecting both the participant’s world and the analyst’s interpretation. Following this, we *searched for connections across experiential statements* to cluster experimental statements into Personal Experimental Themes (PETs). Each cluster received a descriptive title in step five, *naming the PETs and consolidating and organising them in a table*. We reviewed and defined these PETs for each transcript into a comprehensive table, using colour coding to identify themes. The process was *continued and repeated for each case.* Finally, we developed *Group Experimental Themes (GETs)* by analysing patterns across cases and identifying shared and unique aspects among participants’ experiences. ASA made the initial analysis but collaborated closely with ASD throughout the whole analysis process. ASD had read all the transcribed material and participated in Focus Group 2.

The analysis of data from the two workshops was based on participants’ inputs during discussions, as well as sticky notes and drawings, and detailed field notes taken by ASA and KM in Workshop 1 and Workshop 2, respectively. Workshop 1 was analysed at the group level rather than at the individual level, as it was not possible to isolate individual participants’ contributions. Participants first worked in mono-professional groups to discuss their profession’s view and thereafter in cross-sectoral groups to reach a common solution across sectors. The material from the mono-professional groups enabled an analysis of differences and patterns across professional backgrounds. The cross-sectoral groups provided a basis for extracting and analysing participants’ collective contributions to proposals for an intervention. Workshop 2 was also conducted at the group level. However, the small number of participants made it possible to distinguish individual voices, which were, however, very concordant.

The participants’ ideas for the intervention were gathered from three data sources – interviews, focus groups, and workshops. According to the co-design process, which is inherently iterative, they were integrated to reveal challenges and develop and combine ideas across the datasets. The fieldwork observations were not directly included in the primary analysis, but they provided valuable insights into the context, professionals’ daily work, patient interactions, and cross-sector collaboration.

## 3 Results

The analysis revealed four main themes: 1) Insufficient communication from mental health services caused challenges for GPs in the handover phase, 2) Different goals between professionals resulted in mutual mistrust and blame, 3) Patients felt left on their own after discharge due to a lack of coordination and support services, and 4) Ideas for improving cross-sectoral collaboration. In the following, these themes are presented in chronological order and illustrated with verbatim quotations.

### 3.1 Insufficient communication from mental health services caused challenges for GPs in the handover phase

All participants reported that there was little direct interaction between the sectors. During the patients’ treatment in mental health services, communication was exclusively indirect through the patient. MHPs could advise patients to contact their GP if they experience symptoms of somatic illness:

“…*we tell the patient they should see their GP if they experience dizziness, high blood pressure, or*…” (psychiatric nurse 1, individual interview).

However, when patients received treatment in mental health services for an extended period, GPs expressed a need to be better informed about their patients’ condition and treatment. In addition, GPs experienced that the discharge letters from mental health services frequently lacked follow-up plans, especially regarding pharmacological treatment. They included a recommendation to refer the patient to a practising psychiatrist, which, however, posed challenges due to the long waiting times.

In the cross-sectoral workshop, psychiatrists reported that they were unaware that GPs wanted ongoing information from them. They also expressed surprise that GPs had time to read such information regularly:

“*Do you have time to read such information during your busy day?*” (Psychiatrist 3, Workshop 1).

Furthermore, one psychiatrist asked if it was possible to send information directly to GPs, to which a GP responded that there is a button in the health IT-system labelled “send to own GP”. This indicates a lack of awareness among psychiatrists regarding GPs’ desire and need for ongoing information.

During the patient’s treatment in mental health services, MHPs did not consider interaction with the other sectors as necessary. MHPs viewed their role as treating patients for a time-limited period to alleviate their depressive symptoms sufficiently for them to be transferred to general practice. Nevertheless, MHPs recognised that patients were often not fully recovered at the time of discharge:

*“They have some residual symptoms or challenging life circumstances that continue to make it difficult for them”* (psychiatric nurse 1, individual interview).

Even so, MPHs considered their duty as finished. GPs, however, felt inadequately prepared for managing this handover of patients with complex mental problems. They emphasised that they had to cover many specialities and were not specialists in complex mental health disorders. They often felt left with responsibilities that exceeded their competencies and with no opportunities for professional support from mental health services:

*“We are not psychiatrists, we do a lot, I think. We must be somewhat specialists in everything, … I can do many other things. But this aspect, I find a bit challenging”* (GP3, individual interview).

GPs reported feeling especially uncomfortable managing or adjusting pharmacological treatments due to their unfamiliarity with them. They said they could have supporting conversations with patients and viewed the ongoing contact as beneficial:

*“Because then there is always someone, they [the patients] can turn to”* (GP2, Focus Group 2).

They did, however, express a strong need for closer collaboration with mental health services, because they required expertise from the secondary sector when cases exceeded their competencies:

*“We complete 90% of our patients in the practice. When we refer to another speciality, it is because it is beyond our capacity. We expect specialists to manage and treat the patient until we can take over again, or to receive a clear plan for the patient. It should not be a situation where treatment [in secondary care] ends after a year without any further plan”* (GP2, Focus Group 2).

The GPs said that collaboration with mental health services differed significantly from collaboration with somatic care. Somatic departments provided advice when necessary. In contrast, the GPs often found no opportunity for communication with psychiatric specialists, and their referrals to mental health services were frequently rejected. They felt it was necessary to use specific phrasing to ensure acceptance of referrals. One GP said:

*“It is almost as if you need a PhD in writing referrals to get them accepted”* (GP3, individual interview).

The observation at the jobcentre revealed that the CWs felt ill-equipped to manage citizens with mental health conditions. They said that they lacked knowledge of diseases, including how depression impacted daily life. The CWs had diverse educational backgrounds. Many held degrees in the humanities with no education about diseases. They said that they struggled to understand what GPs and psychiatrists wrote. They expressed a strong desire for dialogue with health professionals, particularly MHPs, to reduce their responsibility for managing citizens with severe depression:

*“It’s a big responsibility for us to bear. And that is why we ask the physicians [asking for written status reports] from time to time…”* (CW3, Focus Group 1).

### 3.2 Different goals between professionals resulted in mutual mistrust and blame

Participants from various sectors expressed divergent goals for the patients. MPHs focused on treating symptoms within a time-limited period, whereas GPs had a more holistic perspective that involved patients’ overall functioning. CWs’ goal was to get patients back to work, and they expressed frustration when MHPs, through written statements, tried to delay employment-related activities because they did not find patients to be ready for work:

*“Our goal is to get them back to work because we see that if they are away from the workplace for too long, everything starts falling apart”* (CW2, Focus Group 1).

The professionals from different sectors often expressed a lack of understanding of each other’s roles, characterised by mutual mistrust and blame. Particularly, GPs and MHPs blamed the jobcentre for stressing the patients and worsening their condition:

*“I have had patients who have experienced full-blown anxiety attacks after leaving meetings with CWs at the jobcentre, and I have observed that patients develop a lot of worrying thoughts that maintain anxiety and depression when they have to think about financial issues and the pressure from the jobcentre”* (psychiatric nurse 2, individual interview).

CWs acknowledged that their system could sometimes be too rigid due to legal requirements, which could lead to mistrust. However, they also perceived that MHPs and GPs might unintentionally reinforce patients’ mistrust of the jobcentre, and they considered that getting the citizens back to work formed an essential part of the treatment of depression:

*“If you tell us that it would be very detrimental for this person to start any work-related activities … it would be the same as telling you to stop the medication you have planned for the citizen”* (CW2, Focus Group 1).

CWs expressed frustration at not being understood, respected for their work, or involved in the treatment process by MHPs. They felt that health professionals viewed them as evil people, only focused on getting patients back to work as quickly as possible without regard for their health. CWs experienced that the jobcentre and the healthcare system operated in two separate worlds, speaking different languages, and working with different goals rather than aligning in the same direction.

*“ … we are in two different worlds, in a way. We speak different languages with citizens … so to say; How can collaboration even be achieved?”* (CW2, Focus Group 1).

CWs believed that their relationship with the citizens might be better than that of the GP. They said that they had better insight into citizens’ needs and offered more thorough follow-up than GPs because they had more time with them and possibly a better relationship:

*“ Sometimes we see them differently than GPs do. GPs might only see them for 10 minutes … and if things aren’t going well, they may suggest trying antidepressants. But when we talk to them every four weeks, and if someone is struggling and doesn’t have a good relationship with their GP, they may not visit the GP very often”* (CW1, Focus Group 1).

GPs, on the other hand, found that they were met with disrespect by the jobcentre when they were asked to rewrite their status reports. They said it was often difficult to grasp precisely what was asked about, which led to repeated inquiries and replies, resulting in irritation and delay of the process:

*“I have a patient with depression following a loss. I wrote that she has depression and grief, which is why she is on sick leave…. The jobcentre then writes back asking for more details about her grief and depression, like I should write that she is crying all the time. It seems that this causes delay and increases anxiety for patients if the municipality does not believe them. It is almost as if patients are suspected of trying to deceive the system”* (GP3, individual interview).

CWs found that MHPs deliberately responded too slowly to status requests, necessitating repeated requests. MPHs did, however, find that their views were disregarded and therefore considered the requests as needless because the jobcentre was not required to follow their recommendations.

*“The municipality makes the decisions, but they ask us for a status report. They often explicitly state that it is not our role to advise the municipality on what kind of financial support the patient should receive; that decision lies with the municipality”* (psychiatrist, individual interview).

### 3.3 Patients felt left on their own after discharge due to a lack of coordination and support services

Patients reported having benefited from offers at the outpatient mental health clinic. However, none of these activities were available after discharge, and they said that GPs had no information about their content. After discharge from outpatient mental health services, the patients felt alone and did not experience any continuous support. They thought they were *“discharged to nothing”*, and that they *“were themselves responsible for their recovery and for reaching out for help.”* Two patients had experienced that their GP was unwilling to make a referral to a practising psychiatrist and instead had been referred to a psychologist, involving a user’s fee. The patients expressed a desire for more communication between general practice and mental health services to avoid misunderstandings.

The patients mentioned that both during and after their outpatient treatment, the jobcentre had contacted them and pressured them to return to work, which they did not feel ready to do at that time in their recovery. It had been stressful to be summoned to attend meetings. Often, they felt misunderstood or not taken seriously by the CWs, who they felt were not sufficiently informed about their mental health. They experienced that CWs lacked the necessary knowledge about depression to support them. The patients felt pressured by the jobcentre’s goal for them *“to return to work”*, which was contrary to their own goal *“to have a good life”*.

### 3.4 Ideas for improving cross-sectoral collaboration

GPs and CWs expressed a strong need for a more structured collaboration on this patient group, especially in the follow-up phase after discharge from a mental health clinic. In the workshop, they emphasised the importance of information sharing in the handover phase at discharge. They proposed an online meeting just before discharge with the patient, the MHPs, the GP, and CW, who would follow the patient after discharge to plan follow-up and arrange the first meetings with relevant professionals after discharge. They believed that this would alleviate patients’ concerns and reassure them that they were in safe hands after discharge.

For the follow-up phase, the participants proposed a model in which a mental health clinician acts as a bridge-builder between the sectors to improve care and collaboration. Ideally, this clinician should be a psychiatric nurse with access to a psychiatrist for support. The role of the psychiatric nurse should involve conducting assessments and consultations with patients in general practice when needed for at least one year after discharge, assisting GPs with psychiatric inquiries, addressing concerns about patients’ progress or signs of relapse, and ensuring a cohesive and effective treatment process across sectors. The model described by the participants mirrored earlier models of collaborative care with a psychiatric nurse acting as a bridge-building care manager. A psychiatric nurse emphasised the potential advantages:

*“They [GPs] see patients we manage – before or after their treatment in psychiatry, right? Therefore, allocating resources, time, and space for a therapist to be part of their practice would be very, very important”* (psychiatric nurse 3, individual interview).

CWs stressed the need for integrating the employment and treatment system in a collaborative approach and for recognising employment as an essential part of the overall treatment process.

Some GPs had participated in an earlier collaborative care project for patients with moderate depression, who had been treated in general practice. They were delighted with this model and thought that the system could also be extended to cover patients who were discharged from mental health services after treatment for severe depression, and that this would lead to better recovery and fewer readmissions:

“She [the care manager] had much more time to talk with the patients than we did; they were happy with what they experienced with her compared to us. So, we were pleased about it, and it meant we did not need to refer as many patients [to psychiatry] (GP3, individual interview).

GPs perceived that patients with mental disorders were not offered the same quality of treatment as patients with somatic diseases. They mentioned that patients with severe somatic conditions were followed for long periods in secondary care after their treatment had ended. In contrast, patients with severe depression were often discharged without any follow-up:

*“Cancer patients are followed for five years, but patients who have been treated for severe depression are discharged without follow-up, although the condition is just as lethal”* (GP5, Workshop 1).

Patients agreed with the proposals from the health professionals and CWs about having a care manager with experience in psychiatry, preferably a psychiatric nurse. They would like to be introduced to the care manager at the end of the outpatient treatment to build a relationship before discharge. However, patients also felt a need to monitor their condition in a smartphone application (app), which the care manager could access and respond to if there were signs of relapse or suicidal thoughts. The patients stated that they were more vulnerable after discharge than perhaps acknowledged in primary care and would not always have the energy or initiative to contact health professionals themselves if their condition worsened.

## 4 Discussion

This study found that collaboration between mental health services, general practice, and the municipal jobcentre about patients with severe depression was impeded by divergent goals and priorities and by professionals’ limited knowledge of each other’s responsibilities and needs for collaboration. The professionals’ limited understanding of each other’s roles, combined with their communication style through written forms, could lead to misunderstandings, mutual mistrust, and blame. GPs and CWs received no information from mental health services during patients’ treatment there, and discharge letters to GPs often lacked detailed follow-up plans and advice about patients’ future pharmacological treatment. CWs felt that they lacked knowledge of mental disorders and needed opportunities for direct contact with MHPs.

Health professionals and patients experienced that CWs at the jobcentre pressured patients to return to work too quickly, while the CWs viewed health professionals as unnecessarily delaying this return. All participants agreed on the need for developing a more structured collaboration, suggesting a collaborative model with a care manager to bridge the gaps. Patients stressed that they were still vulnerable after discharge and that an app would be helpful to monitor their symptoms so they could be contacted at signs of relapse or suicidal thoughts.

Following our findings, other studies have shown that although participants express an interest in cross-sectoral collaboration, this can be hindered by a lack of oral communication and limited mutual understanding of competencies, roles, and goals (47–55). Whereas MHPs had limited need for contributions from the other sectors in their work, GPs were dependent on adequate discharge letters. They requested more opportunities for professional support from mental health services.

Studies have shown that limited cross-sectoral collaboration between mental health services, general practice, and municipalities is a result of a combination of factors, including different professional cultures, communication barriers, and structural challenges (8, 56, 57). These factors can lead to fragmented care and negatively impact patient outcomes (56).

Both GPs og CWs wanted ongoing information from MHPs, but the MHPs were unaware that they needed or had time to read it. A 2012 study (58) highlighted that GPs often feel they work in isolation, and strongly support improving sparring with MHPs. Support from psychiatrists is essential for GPs to manage complex psychiatric cases (58). Similarly, GPs in our study expressed feeling insufficiently competent to treat severe depression and missed having direct access to specialists for consultation, particularly regarding pharmacological treatment.

Another Danish study showed that GPs and MHPs shared views on their collaboration (17). Strengths included joint consultations and advice on treatment dilemmas, while limitations involved limited opportunities for meetings and knowledge sharing, which aligns with our findings. Both groups agreed that collaboration could be improved through the sharing of information, direct phone lines, and scheduled times for phone consultations (17).

Additionally, CWs were dependent on knowledge from and information sharing with MHPs and GPs, but CWs experienced that these needs were not sufficiently met. Similar findings have been reported in other studies, with variations in health professionals’ and CWs’ motivation to engage in collaboration (55, 59, 60). In line with other studies (47, 60, 61) and with resource dependency theory, the level of dependency on resources (knowledge, skills, finances, or materials) from other organisations strongly influences collaborative relations among organisations, also in health and social care (61, 62). Thus, while the work of the GPs and the CWs was dependent on information from MHPs, the reverse was not true, reflecting an asymmetry in dependence. This asymmetry influenced the perceived need for collaboration, with MHPs expressing the least need.

CWs and health professionals displayed a mutual accusing attitude and mistrust against each other, and similar collaborative problems have been reported elsewhere, also leading to delays in treatment (55).

According to Gittell et al.’s relational coordination theory (63), task integration relies on a mutually reinforcing process of effective communication and strong relational ties. Experiences of mistrust and disrespect can create a negative cycle of misunderstandings and infrequent or inaccurate communication, further limiting knowledge sharing and leaving professionals with limited insight into how to support one another. Gittell et al. state that shared goals, shared knowledge, and mutual respect, supported by frequent, timely, accurate, problem-solving communication, are essential for effective collaboration, and enabling professionals across sectors to coordinate their work more effectively (63), but achieving this is often difficult (64). In our study, communication between sectors was sparse and formal (in writing), with few opportunities for direct oral communication and limited conveyance of treatment plans from mental health services to GPs. Furthermore, the professionals had differing goals and perspectives regarding how quickly patients should return to work, which created tensions among them. Low-quality relationships undermined the quality of communication and hindered professionals’ ability to coordinate their work effectively. A study on chronic widespread pain likewise found that GPs tended to extend the duration of patient treatment before return to work, while CWs aimed to shorten it. The study concluded that these differences, along with inadequate communication and limited understanding, harmed collaboration (47).

A scoping review by Tomaschek et al. (54) summarised role distributions and components in cross-sectoral collaboration that have potential for improving care of patients with complex chronic conditions. The authors identified several key interventions, including clear role definition, the provision of resources for knowledge transfer, and education from specialists. The review demonstrated that GPs can “take on additional responsibilities successfully with streamlined specialist support” [46, p.7], which was what the GPs in our study asked for. Generally, the review found that improving GP-specialist collaboration increases both provider and patient satisfaction, as well as health outcomes (54). The review only addressed somatic illness, but the results are in line with a Danish study, which showed that GPs felt they lacked support from psychiatrists in managing mental health conditions and were often left with problems exceeding their competencies (8), which was also the case in the present study.

The patients felt left in limbo when discharged from mental health services, having to find their way in the system, which did not recognise how vulnerable they still were. Both patients, health professionals, and CWs asked for a focus on the handover phase and a collaborative approach to support patients for an extended period after discharge, possibly as an extension of well-known collaborative models for patients with depression treated in general practice and with a care manager as a bridge builder between the sectors, however, also involving the social sector. In a sub-study within the same project as the present article, we conducted individual interviews with 12 patients who had been treated for severe depression at an outpatient secondary mental health clinic (45) – the same clinic from which the patients in this study were recruited. They were interviewed before their discharge from secondary to primary care. The results indicated that patients placed a high value on individualised treatment and expressed concern about losing contact with MHPs after discharge. They suggested tailored treatment, flexible return-to-work solutions, and continued contact with familiar MHPs. The findings emphasised the need for coordinated, patient-centred care that provides patients with continuous and reliable cross-sectoral support, helping to prevent relapse. Previous models of collaborative approaches for depression have shown both effect and patient satisfaction (31, 55, 65). However, to our knowledge, no studies have involved the social sector or integrated users’ perspectives through a co-design process, which would make such models more complex.

### 4.1 Strengths and limitations

This study has several limitations. The results are based on fieldwork observations, interviews, focus groups, and workshops with health professionals and CWs from a few organisations and patients in a single region in Denmark. The study predominantly covered urban areas, and collaborative relations may differ in more remote areas with fewer mental health specialists, where patients might rely more on their GP for continued care. However, the participants in this study also stressed the long waiting times for practising psychiatrists, and this problem can be assumed to be even worse in other areas. It is also more challenging to hire CWs in more remote areas, which might result in a larger caseload and less time for the citizens.

The iterative process of combining data collection and analysis allowed the researchers to refine their questions and focus areas throughout the study, thereby enhancing the depth and reflexivity of the findings.

Four patients participated in Workshop 2. Five had initially agreed to participate, but one cancelled on the day, as they felt too vulnerable to participate. As previously described, 12 patients were interviewed in a sub-study (45) within the same project, and their views on post-discharge treatment were consistent with those of the patients included in the present study. The study’s strength lies in the multiple data collection methods, which we consider provided information power (66). However, the study included only a small number of patients, which constitutes a limitation. This limitation was partly compensated for by drawing on results from a previous interview-based sub-study with patients (45), in which other patients raised the same critical comments and proposals that emerged in Workshop 2 from the same clinic.

Although limited in scope, its findings align with existing evidence, and we think the results are transferable to other areas in Denmark and similar healthcare systems.

## 5 Conclusion

This study identified significant relational and structural barriers that hindered effective cross-sector collaboration during the transition of patients with severe depression from specialised outpatient secondary mental health services to continued treatment and support in primary care.

Cross-sector collaboration was hindered by low relational coordination, characterised by differing roles and goals, a lack of knowledge sharing and poor communication. Notably, professionals and patients held different views on the focus and approach to treatment and support across sectors, including differing opinions on the appropriate timing for returning to work. This contributed to weakened collaboration, which was further exacerbated by an asymmetry in dependence between sectors. GPs and CWs were dependent on information from MHPs – a dependency that was not equally reciprocated. Also, GPs and CWs wanted ongoing information sharing and more detailed discharge letters from psychiatrists, specifically regarding follow-up on medication and ongoing status assessments of when patients could return to work. Psychiatrists were unaware that GPs and CWs had the capacity to receive and review ongoing information updates. Additionally, the three institutions were characterised by distinct professional cultures, which made it challenging to modify established workflows and collaboration practices.

Based on the above, several barriers hindered effective cross-sectoral collaboration. These challenges resulted in fragmented and insufficient continuous support after discharge from outpatient treatment. Consequently, patients were often left to navigate a complex system on their own, increasing their risk of relapse.

Building on these insights, incorporating perspectives from healthcare professionals, CWs, and patients generated valuable ideas on how to improve collaboration and prevent relapse in severe depression. Participants suggested introducing a coordinated liaison or care coordinator – preferably with psychiatric experience – as a bridge-builder between sectors, along with online planning meetings before discharge. Additionally, they recommended enhancing communication channels to enable GPs and CWs to contact MHPs when needed more easily. Patients emphasised feeling vulnerable after discharge and suggested that a symptom-monitoring app could facilitate timely contact in case of relapse. They also requested flexible returning-to-work solutions and ongoing contact with familiar MHPs.

The findings highlight the urgent need for coordinated, integrated, and continuous care to prevent relapse. However, implementing such an approach remains challenging in a system where the three institutions operate in isolation without shared leadership. Consequently, strong political and administrative commitment is essential to drive meaningful change and improve outcomes for patients with severe depression.

## Data Availability

The dataset generated in this qualitative study, which includes fieldwork observations, interviews, focus groups, and workshops, cannot be shared publicly due to ethical and privacy considerations. The data collected involved sensitive information from participants, and in accordance with ethical guidelines and privacy regulations, it is not permissible to make these datasets publicly available. The themes discussed in the article are based on primary data, which have been summarized and presented in the main text of the article. Requests to access the datasets should be directed to Anne Sofie Aggestrup, anne.sofie.aggestrup@regionh.dk.
