# Supplementary material for: Collaborative challenges during transition of patients with severe depression from secondary mental health services to primary care and opportunities for improvement - a qualitative study
Source: Front Psychiatry. 2025 Oct 1;16:1553930. doi: 10.3389/fpsyt.2025.1553930 (PMC12521750; doi:10.3389/fpsyt.2025.1553930)
Supplement: Supplementary file 1 [file DataSheet1.docx]

| **Interviewspørgsmål** | **Tema** |
| --- | --- |
| 1. Hvor meget fylder patienter med depression i din praksis?  - Hvad er det for et sygdomsbillede, de har? - Har de andre problemer end depression?  1. De fleste patienter med let/moderat depression bliver jo behandlet i almen praksis, men oftest bliver patienter med svær depression indlagt på en psykiatrisk afdeling. Synes du, at patienter med svær depression adskiller sig fra patienter med let/moderat depression?  - Hvis ja, hvorfor og på hvilken måde adskiller de sig fra hinanden?  1. Hvilke udfordringer møder du som behandler, når patienterne kommer tilbage til din praksis fra et behandlingsforløb i psykiatrien?  - Du må gerne fortælle nogle patienthistorier… | *Hverdagen i praksis og med patienter med depression (overordnet)* |
| 1. Jeg har interviewet en patient, som netop er afsluttet fra sit ambulante behandlingsforløb for depression og er kommet tilbage til din praksis. Kan du huske patienten?  - Har du været i kontakt med patienten – hvis ja, kan du huske, hvem der tog kontakt og hvorfor?  1. Hvad tror du, der skal til, for at denne patient (eller patienter generelt) undgår at blive deprimeret igen?  - Hvis har du af redskaber og muligheder til at fortsætte behandlingen? | *Fra IAA til Almen Praksis (parret med patient fra IAA)* |
| 1. Samarbejder du med andre, fx psykiatrien eller jobcentre, når patienterne udskrives fra psykiatrien og skal tilbage til din praksis og i forløbet herefter?  - Er der nogle, du samarbejder mere med end andre? – hvorfor? - Har du nogle erfaringer du vil dele om samarbejdet…? | *Nuværende samarbejde mellem sektorer* |
| 1. Har du nogle idéer til ideelle behandlingsforløb, når patienter med depression afslutter deres behandling fra psykiatrien og skal tilbage til din praksis?  - Hvem skulle være involveret i behandlingen? - Hvis du selv kunne bestemme, hvordan ville du så ønske, at en samarbejdsmodel mellem almen praksis, psykiatrien og kommunens jobcentre så ud? - Ville behandlingen af svær depression i almen praksis kunne indgå i et kronikerkontrol program? - Skulle man lave en pjece om fx søvn og depression? En App?  1. Vi ved, at der er nogle tiltag, som har haft effekt i den ambulante psykiatri (kognitiv adfærdsterapi, psykoedukation, antidepressiv medicin, yoga og motionsgruppe), og vi vil gerne have, at patienterne kan fortsætte deres recovery. Vi vil derfor gerne sammen med almen praksis undersøge, hvad der giver mening, når patienterne fortsætter behandlingen i almen praksis.   Hvad tænker du om det?   - Har I erfaring med den type behandling (som den behandling patienterne får tilbudt i den ambulante psykiatri) i din praksis? | *Den optimale samarbejdsmodel* |
| 1. Er der noget, som du synes er vigtigt, vi får med, som vi ikke er kommet ind på under interviewet?   Mange tak | *Afslutning* |
